# Intersectionality in Medical Education: A Meta-Narrative Review

**DOI:** 10.5334/pme.1161

**Published:** 2023-11-07

**Authors:** Maham Rehman, Divya Santhanam, Javeed Sukhera

**Affiliations:** 1 Michael G. DeGroote School of Medicine, McMaster University, Hamilton, Ontario, Canada; 2 Department of Medicine, University of Toronto, Toronto Ontario, Canada; 3 Institute of Living and Hartford Hospital, Hartford Healthcare Behavioral Health Network, Hartford, CT, United States

## Abstract

**Introduction:**

Despite increasing attention to improving equity, diversity, and inclusion in academic medicine, a theoretically informed perspective to advancing equity is often missing. Intersectionality is a theoretical framework that refers to the study of the dynamic nature of social categories with which an individual identifies and their unique localization within power structures. Intersectionality can be a useful lens to understand and address inequity, however, there is limited literature on intersectionality in the context of medical education. Thus, we explored how intersectionality has been conceptualized and applied in medical education.

**Methods:**

We employed a meta-narrative review, analyzing existing literature on intersectionality theory and frameworks in medical education. Three electronic databases were searched using key terms yielding 32 articles. After, title, abstract and full-text screening 14articles were included. Analysis of articles sought a meaningful synthesis on application of intersectionality theory to medical education.

**Results:**

Existing literature on intersectionality discussesthe role of identity categorization and the relationship between identity, power, and social change. There are contrasting narratives on the practical application of intersectionality to medical education, producing tensions between how intersectionality is understood as theory and how it is translated in practice.

**Discussion:**

A paucity in literature on intersectionality in medical education suggests that there is a risk intersectionality may be understood in a superficial manner and considered a synonym for diversity. Drawing explicit attention to its core tenets of reflexivity, transformational identity, and analysis of power is important to maintain fidelity to how intersectionality is understood in broader critical social science literature.

## Introduction

The concepts of equity, diversity, and inclusion (EDI) have received increasing attention and are highly valued ideas in academic medicine and medical education. A plethora of theories, frameworks and approaches have been applied to better understand how we can improve EDI within healthcare spaces both for patients and healthcare professionals. Such approaches have included cultural competence, cultural humility, and various forms of transformative and critical pedagogies [1,2]. Many aspects of critical theories and frameworks are intrinsically related to the concept of intersectionality. Originating as a fourth wave feminist theory and rooted in Black feminist theory, intersectionality broadly refers to the dynamic and contextually specific ways in which social categorization, power, and prejudice intersect with one another to produce and reproduce systems of oppression [3,4,5]. Intersectionality is a component of Critical Race Theory (CRT) which refers to a legal and analytical framework that examines the ways in which systemic racism is embedded within society and exacerbates inequities [6]. Despite its relevance and increasing interest in the topic, empiric study on how intersectionality is understood and applied in the specific context of medical education is needed [7].

Intersectionality is commonly understood as a concept to be both understood and applied in both theoretical and methodological contexts [8]. As a framework or theory, intersectionality suggests that researchers should consider the intersectional nature of their research topic, while incorporating an analysis on the dynamic influence of overlapping social identities [9]. As a methodology, intersectionality encourages a detailed analysis of a topic or phenomena through the lens of overlapping and dynamic social identities and power relations [9,10]. For example, a research project exploring the efficacy of women’s reproductive health education could use intersectionality as a framework or methodology to consider how individual and structural forces influence how reproductive health is understood and applied in a health education context. As a framework, intersectionality facilitates a nuanced understanding on how various social identities relate to one another in the context of healthcare and health education. As a methodology, intersectionality may facilitate deeper inquiry into how identity categories might influence healthcare and health education.

As intersectionality has evolved, many working definitions have been proposed, which share common features [11,12,13,14]. First is recognition that individuals have multiple identities that are interconnected. Intersectionality requires analyzing multiple social categories simultaneously and cautions against conceptualizing identities as categorical without considering how they relate to one another [15,16,17]. Second is recognition of the role of power, inequality, and oppression in perpetuating inequity. Intersectionality requires theorizing and analyzing how power and inequality are interrelated [5]. Third is recognition that identities are properties of the individual but are also formed and/or influenced by socio-historical contexts [16]. Crenshaw [3] emphasizes in foundational writings on intersectionality that one’s identity and its significance is context-specific and dynamic. Therefore, intersectionality assumes that processes and outcomes of identification are dependent on framing and context [18], and emphasizes the dynamic nature of the formation and maintenance of various social identities [9,17,19,20].

Although research on advancing EDI and social justice is growing, there is a paucity of research on how intersectionality is understood and applied in medical education. There are some calls for improving medical education’s understanding of how intersectionality views social categories in the context of professional identity formation or bias mitigation, yet detailed examples remains scarce [21,22]. In addition to supporting a inclusive approach to professional identity formation, intersectionality may be useful for faculty developers to ensure clinical teachers have a sophisticated understanding of personal and professional identities to support improving EDI related competencies [23]. Intersectionality could also play a role in improving physician-patient communication, and broader structural reforms to advance health justice. Overall, intersectionality is a relevant and timely concept for medical education to consider [16]. An intersectional lens may help address critiques of current approaches in EDI that are often performative, superficial, and lack evidence-informed rigor [24]. Intersectionality may help medical educators and researchers better conceptualize systems of advantage and disadvantage while integrating structural and power analyses. When considered in the context of CRT and post-colonial theory, which emphasize the sociopolitical and economic legacy of imperialism and its consequences intersectionality may be useful to advance work on EDI and health justice in a meaningful and sustainable way.

Given the unique potential for intersectionality to facilitate efforts regarding EDI and health justice in medical education and a paucity of literature on the topic, we sought to explore how intersectionality may be understood and applied by medical educators. By gaining understanding on how it may be both understood and applied, we aim to provide a meaningful synthesis of the literature on intersectionality to inform future efforts to incorporate intersectional methodologies and framework within health education.

## Methods

We chose to conduct a meta-narrative review, which is a practical and theory-informed approach to exploring topics that have been conceptualized and investigated in diverse ways by different disciplines or schools of thought. A meta-narrative review captures contradictions within the literature while seeking to facilitate sense-making of diverging perspectives. The core principles of meta-narrative review as described by the RAMESES international criteria proposed by Wong and colleagues [25] were reflected throughout our review process. These included pragmatism, pluralism, historicity, contestation, and reflexivity.

A meta-narrative approach aligned with our desire to better understand the different ways in which intersectional theory has been conceptualized by researchers while comparing and contrasting the various research traditions that have been used to explore and describe intersectional theory. We considered other forms of literature synthesis such as a critical review or a scoping review, however, given the rapidly evolving literature on EDI in medical education and our desire for a rigorous review within a scarce set of literature, we hoped to track how intersectionality was understood and applied in a range of disciplines over time, aligning with meta-narrative principles. A meta-narrative approach also allowed us the opportunity to explore how narratives regarding intersectionality have evolved and amalgamated with modern discourse on social justice, identity, and diversity. Our specific objective was to explore the ways in which diverging conceptualizations of intersectional theory can be reconciled and understood in the context of medical education.

### Search Strategy

As part of the initial research strategy, the research team met at regular intervals and conducted a preliminary review of the foundational writings on intersectionality and existing literature in medical education. Next, we explored literature on the application of intersectional theory to medical education using meta-narrative methodology. Throughout the process, we compared and contrasted differing narratives on both how intersectionality is understood and applied.

To illuminate our topic from multiple angles and perspectives, we searched three databases (PubMed, SCOPUS and JSTOR) for English-language studies published from January 1, 1989, when intersectionality was first coined by Dr. Crenshaw, to November 2020. We used the following search strategy: ‘intersectionality’ AND ‘health education’ OR ‘medical education’ OR ‘health professions’ OR ‘medicine.’ We reference checked relevant retrieved articles to identify additional publications.

### Study Selection Criteria and Process

In alignment with the principles of meta-narrative synthesis, we considered historicity as part of our process. We included articles if (1) the article was published in or after 1989, the year in which Kimberlé Crenshaw coined the term and published her work on intersectionality. (2) Intersectionality was explicitly defined and discussed and (3) the authors cited Crenshaw. This ensured that the authors attributed Crenshaw’s work and confirmed that they had appraised her foundational writings on the theory. (4) Intersectionality was discussed in connection to medical education, training, or practice. For instance, if an article applied intersectional methodologies to address gaps in nursing education and/or training, it was included; however, if it described intersectional methodologies in psychology research it was not included. We recognize and appreciate the groundbreaking contributions of esteemed Black feminist scholars, such as Patricia Hill Collins and bell hooks, who had discussed the intricate interplay of race, gender, and class before 1989 [26,27]. While acknowledging their valuable insights, our deliberate choice to prioritize Crenshaw’s seminal writings as an inclusion criterion was driven by the intention to foreground the essential work of critical feminist scholars, particularly Black women, whose contributions to the understanding of intersectionality are often appropriated and not sufficiently recognized. Our choice was also consistent with the principle of pragmatism and historicity, as we sought to map the use of specific term intersectionality across time within literature that was relevant to medical education.

Our exclusion criteria was informed by the principle of pragmatism [25], with a clear focus on selecting literature that deepens readers’ understanding and contributes to sense-making within the context of medical learning and education. As a result, we excluded works that did not explicitly link or explore intersectionality in relation to medical learning or education. To gain comprehensive insights into the application of intersectionality in medical education, we specifically excluded articles that failed to incorporate intersectionality in their methods, results, or discussion sections. This decision was consistent with the scope of our review, which aimed to assess the practical implementation and utilization of intersectionality. Additionally, we opted to exclude dissertations, commentaries, and literature reviews since they tend to concentrate on characterizing intersectionality rather than critically applying it in practice.

**Figure 1:**
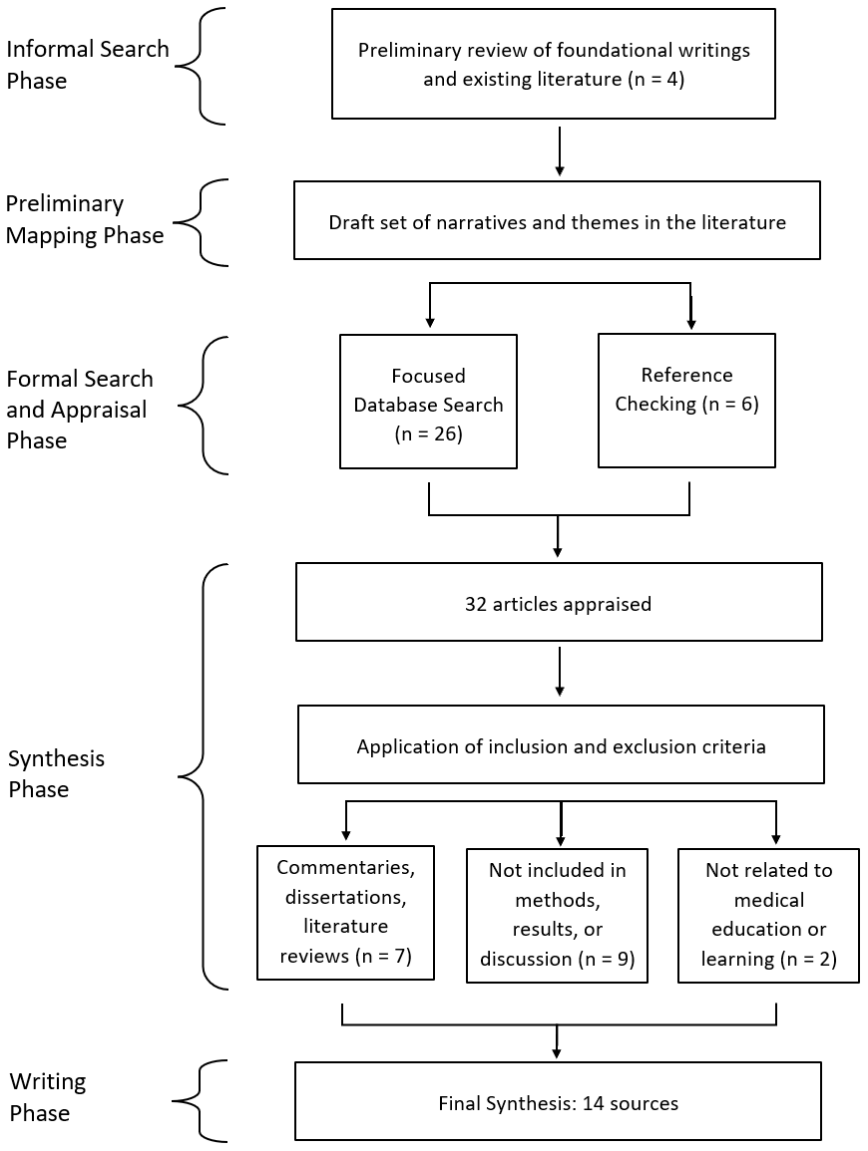
Article selection process.

Database searching identified 32 articles. After an initial review, 26 full-text articles were selected for further screening and analysis. Referencing checking identified additional six articles. A total of 32 articles were reviewed and screened against the inclusion and exclusion criteria. Any conflicts were resolved through discussion and consensus among all three authors. Ultimately, 14 articles were chosen for extraction and analysis. Figure 1 provides a visual representation of our review process (Figure 1).

### Data Extraction and Analysis

Our team consisted of the first author (M.R.), a third-year medical student, a first year internal medicine resident (D.R.S.), and a practicing physician, faculty, and PhD in health professions education (J.S.). The first author reviewed each article with 1 other team member reviewing and extracting data. Extraction collected data on (1) the terms used to define and describe intersectionality; (2) problematization of the research; (3) methods used; (4) application of intersectionality to health professions education. Data extraction from each source also included: author(s), year, and connection to other theory and theorists.

Our analytic framework included a series of questions to explore how intersectionality was understood and how it was applied within each literature source. We also explored why intersectionality was evoked, how it was problematized, and attempted to better understand the epistemological positioning of intersectionality by the authors. In this context, epistemology refers to assumptions about the nature of knowledge. Throughout the process, the team engaged in iterative meetings to exchange perspectives on the articles and scrutinize how the findings related to historical and theoretical approaches concerning the application of intersectionality. In our initial analysis, we identified recurrent concepts and ideas that resurfaced during our discussions. These key concepts were then thoroughly examined by revisiting the literature to elucidate them further.

### Reflexivity

Reflexivity is an essential component of intersectionality. Therefore, the research team consistenly sought to understand and consider how the theory of intersectionality applied to the self, community, and systems. In the context of this research team, all members are people of color who have experienced racialization, are wary of the intersections of race, identity, and experience, and can personally connect to Crenshaw’s writings. At the time this review was initially written, the first author was a pre-health professions student, the second author was a learner, and the senior author was a practicing physician and PhD research scientist in medical education. Thus, it was highly important for us to be cognizant of our identities and experiences and how that may influence our analysis of intersectionality. Therefore, throughout our collaboration we were explicit in our assumptions and experiences. Our discussions at every step of the review process incorporated reflection and critical consciousness. For instance, one of our analytic discussions on the connections between power and intersectionality required us to consider our own unique, dynamic, and overlapping identities and how they relate to power relations in medical education and within our team itself.

## Results

Our meta-narrative synthesis findings suggested that intersectionality in medical education was described in both theory and practice in medical education. We identified cross cutting meta-narratives (See Table 1) that allowed us to highlight connections and contestations on the ways that intersectionality was framed and understood between and among each narrative thread. We found that intersectionality was positioned as relevant to research, innovation, and teaching practices. Although intersectionality was often described as a useful tool to explore the intricate relationship between identity and categorization, it was also invoked in diverse ways, such as through fostering investigations into systems of power, evaluations of medical education’s impact on identity development, and driving transformative change towards a more inclusive and less oppressive future. The central narrative tension in our findings related to the tension between how intersectionality is described in theory versus in practice.

**Table 1:** Cross Cutting Meta-Narratives.


TITLE OF META-NARRATIVE	DESCRIPTION	NUMBER OF SOURCES^a^

Competency and Understanding	Intersectionality as a tool to enrich understanding of cultural competency and categorization	6

Power and Identity	Intersectionality as a tool to foster self-reflection on interconnections between identity, experiences, and systems of power	3

Structural Change	Intersectionality as a tool to remediate structural barriers at the healthcare institutional and policy levels	5


^a^Number of sources from final synthesis.

### Meta-Narratives on Defining and Conceptualizing Intersectionality

Overall, the articles in our analysis appeared to conceptualize intersectionality in relation to three primary concepts, (1) the complexity of identity formation and lived or living experiences, (2) the mutually transformative relationship between multiple identities and the social contexts they are situated in, (3) the greater systems of power that identity is contextualized within material contexts. For example, Muntinga and colleagues [28] described intersectionality as coexisting and mutually reinforcing social identities that vary depending on location, while Eckstrand and colleagues [29] idefined intersectionality as the interconnected nature of social categorizations and how they are applied to different populations. When defining intersectionality, most authors described a complex interrelationship between identity and social context while noting how both factors produce experiences of advantage and disadvantage for certain social identities. For medical educators, intersectionality was understood as an approach to identifying and understanding the identities embodied by medical learners and how such identities related to their professional development and clinical experiences.

### Meta-Narratives on How Intersectionality Relates to Social Categorization

While numerous authors presented comprehensive definitions of intersectionality and its facets, our review uncovered significant variations in how these authors understood and interpreted the theory. Meta-narrative synthesis affords an opportunity to understand various contradictions and make sense of how such contradictions may relate to historical and epistemological contexts. Varying narratives were particularly salient in how intersectionality was understood and interpreted in relation to social categorization. Categorization refers to the process of grouping similar experiences and/or identities. Both meta-narratives appear consistent with and in alignment with seminal literature on the topic.

One meta-narrative suggested that intersectionality could be utilised as a tool to understand and explore *how* various “categories of identity” relate to one another [22,30]. For example, a healthcare professional could utilize intersectional theory to examine their multiple statuses, whether it be via social class, gender or race, and then further analyse the relationships between these social categories. They could then similarly analyse the various overlapping identities of their patients and thus better understand their own relationship with them. Muntinga and colleagues shared the view that intersectional theory utilized a multiple category approach, emphasizing that examining their interrelations transcended single categories of difference [28].

A distinct meta-narrative suggested that intersectionality could facilitate *how* social categorization was driven through power asymmetries and oppressive dynamics over time. Scholars argued that intersectionality provides a way to adapt competency approaches with complexity in mind, allowing for a more nuanced approach to understand the lived experiences and oppression that goes beyond existing categorizations in medical education [31]. For example, Monrouxe stated that it was most important to examine the social, political and historical processes by which identity was formed [16]. Thus, it was evident that intersectionality can be understood as a lens to better view the categorization of identity or as a mechanism to transcend fixed conceptualizations of identity itself.

### Understanding Identity, Power, Driving Change: Multifaceted Purposes

Narratives on how intersectionality was applied were diverse and varied, yet complementary. Some used intersectionality as a mechanism to foster critical self-reflection and identity development, others as a tool to understand how structural inequities shape discriminatory practices, and others invoked the utility of intersectionality as being applied through education and training to address inequity through action.

Several authors understood intersectionality as a mechanism to engage in self-reflection and foster identity development among trainees. For example, Potter used intersectionality to increase students’ self-understanding and perception; encouraging reflection on who they are and the experiences that have shaped them [32]. Similarly, Monrouxe applied intersectionality to frame identity development among medical students, recognizing it as a framework to view the self, others, and the environment [16].

Intersectionality was also used as a tool to understand how education and healthcare systems shaped discriminatory practices in dynamic and multifaceted ways. In particular, certain narratives regarding intersectionality emphasized that discrimination happens through transformative rather than additive mechanisms. For example, Tsouroufli interrogated and examined power structures through the lens of intersectionality in conjunction with critical theory [10]. This included exploration of social categories including gender, race, ethnicity and their effect in systems, languages and institutions. Blackie and colleagues [31] built upon this idea to employ the concept of narrative intersectionality, which includes storytelling that enables medical student’s understanding and evaluation of different identities in relation to inequality and social disadvantage. Several articles also invoked facets of intersectionality in relation to power to inform an improved historical and structural analysis of social inequities [16,33]. For instance, Paradis and colleagues [33] recognized intersectionality as a means to examine power structures through various social contexts such as language, structures, and organizations.

Some authors viewed intersectionality as a tool for both self-reflection and education. For example, intersectionality was considered a mechanism that could facilitate or sensitize approaches to self-reflection as learners critically reflect on their assumptions and biases. Eckstrand and colleagues described intersectionality as a mechanism to identify the biases that underpin social disadvantage [29]. Such authors often stated that intersectional frameworks allowed individuals and institutions to understand the ways in which historical injustices have translated to ongoing discriminatory practices. This identification can then be mitigated through interventions. In application of this idea, Wilson and colleagues sought to decrease implicit bias in patient-healthcare provider interaction through using intersectionality as a preliminary reflection exercise [34].

### Tensions between how intersectionality is understood and applied

Our analysis of the diverging ways in which intersectionality may be understood and applied suggested a tension between how intersectionality is conceptualized or applied in theory versus practice. We use the term practice to mean the process by which the term is applied and embodied in medical education. Although variation in regard to the facets of intersectionality was beneficial for advancing discourse, too much variation produced gaps in understanding and application. It is commonly acknowledged that a disconnect often exists between theory and practice, leading to divergent understandings of concepts and their application. This phenomenon is observable across various disciplines within medical education scholarship. However, it is essential to recognize that the utilization of intersectionality as a concept or theory does not conform to this pattern. On the contrary, the persistent gap between the theory of intersectionality and its practical implementation can result in harmful consequences for equity-deserving groups.

We found that narratives regarding intersectionality often foregrounded intersectionality as a theory, described its core tenets, its purposes, and its outcomes, yet lacked the sufficient depth and specific process or mechanisms through which intersectionality could be translated from theory to practice. The first example of this meta-narrative tension was through over-simplification. Some authors mentioned the term intersectionality without any specific methodological, conceptual, or theoretical framework to understand or apply intersectionality into medical education. For example, Raj and colleagues [30] explored data on gender parity and intersectionality in medical education by evaluating gender norms within learning spaces. While they mentioned that intersectionality involved multiple forms of social marginalizations simultaneously (e.g. sex and race), their final analysis stated that women of colour face further underrepresentation in academic medicine spaces. Although their recommendations called for institutional change, the authors did not delve into the mechanisms by which multiple forms of social marginalizations occurred simultaneously and subsequent effects on gender parity outcomes. The reasons *why* woman of colour face further underrepresentation in academic medicine, *how* this was embodied in greater power structures and what policies would specifically address the unique barriers woman of colour face in academic medicine were not outlined. Thus, even though the authors delved into discussions on social categorization and transformative advocacy, their work displayed a notable deficiency in reflexive analysis. The gap in their theoretical framework becomes evident when we examine the absence of a comprehensive exploration of the overarching systems of power that influence the multiple dimensions of identity. This deficiency not only exists in theory but also extended to the practical application, where the concept of intersectionality was introduced as a means to acknowledge greater diversity within identity constructs but was not rigorously applied when examining real-world data.

The second example was a meta-narrative of dilution. Some papers combined intersectionality with other educational frameworks without describing or explaining how such concepts related to one another and may be distinct yet interrelated. For instance, Blackie and colleagues [31] proposed the idea of narrative intersectionality - combining storytelling and identity to appreciate the complexity of patients’ context. They urged medical learners to move beyond enforcing systems of categorization to embracing the singularity and uniqueness of individual identity. Similarly, Jones and colleagues [35] highlighted that intersectionality helped learners understand the complexity of patient identity. They described that categorization in medicine has applied a ubiquitous lens, citing that labels such as “mens health” or “urban medicine” are over simplified and have failed to consider how categories such as race, ability, and class are factored into health interactions. However, the authors did not provide an approach on how to attain such integrative thinking, once again failing to apply a reflexive lens. For example, *how* does a learner undertsand and appreciate which social categories are relevant during a health interaction. *How* do they apply this learning and what are the implications?

Ultimately, we observed a discussion of social categorization, power dynamics, and transformative advocacy in the literature pertaining to intersectionality. However, there appeared to be a relative scarcity of content pertaining to reflexivity. Consequently, a critical theoretical insight emerging from our research underscores the imperative of incorporating intersectionality into the discourse surrounding reflexivity.

## Discussion

Overall, our review found variation on how intersectionality is understood and applied in medical education. Authors varied in how they understood the purpose of intersectionality, how intersectionality applied to the concept of categorization, and gaps on how to translate intersectionality into practice. Our findings have important implications for medical education scholars, educators, and learners who seek to apply intersectionality towards advancing broader efforts regarding EDI.

Aside from the availability of literature, we attempted to make sense of the narratives on intersectionality in medical education. In general, the included studies understanding and application of intersectionality in medical education has advanced over time, yet perhaps not as far as we would have expected. We found that various scholars differ in the ways in which they emphasize facets of the term. Some scholars emphasized reflexivity as a core component of the theory while others centred explorations of identity and systems of power. Although there is flexibility in interpretation, intersectionality was initially described as a concept that must be understood as an amalgamation of these ideologies [3]. Crenshaw [3] herself urges intersectionality requires understanding “how identities and power work together from one context to another”. Simply, focusing on one facet leads to a superficial understanding on intersectionality. For example, by focusing on the role of identity, intersectional theory often gets equated with “diversity.” This is a harmful interpretation as diversity and intersectionality are separate terms encompassing individual and sometimes overlapping concerns. At the same time, a strict or myopic focus on intersectionality alone fails to consider other race and identity-based theories that can complement an intersectional approach. In our review we found that many authors struggled to articulate the purpose for invoking intersectionality as compared to other theories such as critical race theory and post-colonial theory. It is essential to consider the limits of intersectional theory in explaining social phenomena and how other theories may enrich our understanding of the mechanisms that create social inequality.

Our findings suggest that intersectionality may be understood and applied in a largely superficial way in medical education. This finding highlights the importance of understanding the historical context in which intersectionality was developed, and a more in depth understanding of how it related to other ideas in critical social sciences. Kimberlé Crénshaw conceptualized the term to explain social injustice and the unique marginalization experienced by Black women. Understanding and applying intersectionality to medical education must centre concepts of historical and structural oppression and go beyond buzzwords such as multiculturalism, cultural competency, and diversity. Rather, it should be recognized as an approach to dismantle systems of social inequality.

### Moving Forward: Implications

Without effective translation from theory to practice and clear purpose, the incorporation of intersectionality in medical education research becomes diluted. Moving forward, we must ask ourselves how we can meaningfully integrate intersectionality in medical education. This requires considering two main implications. First, a deeper understanding of intersectionality and its theoretical components is needed. Second, reflexivity can be a helpful tool in facilicating application of intersectionality.

#### Deeper Understanding

**Figure 2:**
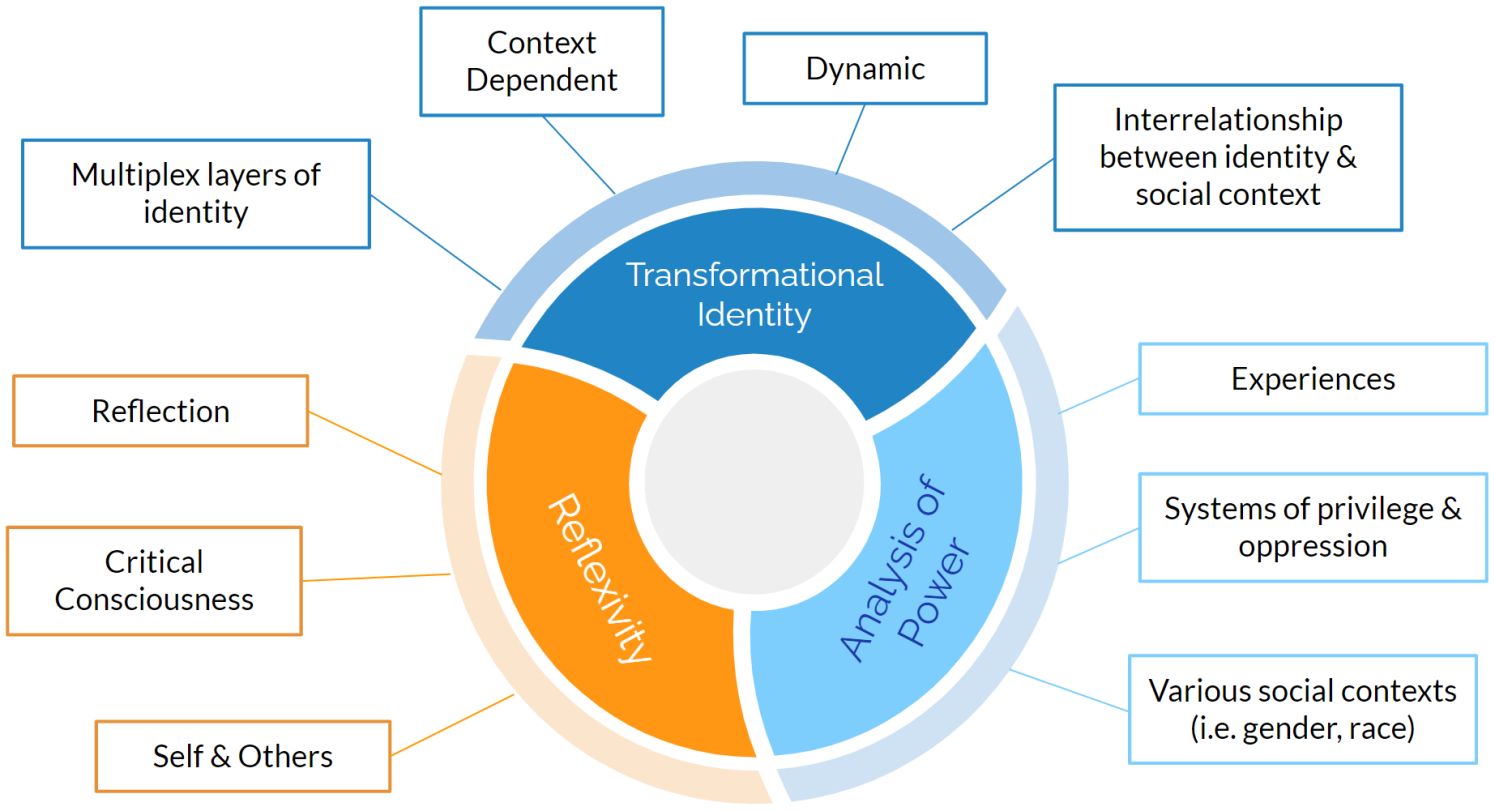
Core tenants and considerations of intersectionality theory.

Our review suggests that a key first step for medical educators to consider is how to recognize intersectionality in relation to its core tenets of reflexivity, transformational identity, and analysis of power (See Figure 2). Doing so requires exploring the facets of each tenet and how they relate to a medical education context. In other words, before we can critically appraise and apply intersectionality in medical education, we must first understand the fundamental components of the theory.

Reflexivity refers to conscious awareness on the influence of identity and power differentials; it requires an individual to reflect on their privileges, identities, and relationships [36]. It fosters critical reflection to help identify assumptions, beliefs, and potential biases with respect to increasing self-awareness [36]. Reflexivity can be applied at the level of the individual, system, and methodology [28,37].

The epistemological underpinnings of concepts such as reflexivity and intersectionality are intrinsically related to one another. Intersectionality helps us understand identity, inequality, and power while reflexivity prompts reflection on these ideas. In the context of medical education, reflexivity must be fostered among learners through practicing reflection and critical consciousness. This can be achieved through reflexive practices such as examination of individual identities, acknowledging and interrogating assumptions and biases, and analysis of socio-political, cultural, and historical contexts within the setting of the learner [38]. These exercises provide learners greater clarity on how their identity as medical leaders influences the way they interact with the healthcare environment. Specifically, it can prompt identification of biases, accountability for their behaviour, and awareness of disadvantage.

Aside from reflexivity, identity is also a central tenet of intersectionality. Understanding identity and its facets is key to grounding intersectionality in systems of advantage and disadvantage. Identity is understood as a social category in which an individual claims membership. It is the property of an individual but is also formed and/or influenced by socio-historical contexts [16]. Identities are not discrete or monolithic, rather they are relationally defined [17]. In medical education, the idea of identity as dynamic and context-dependent must be reconciled with the use of intersectionality to determine the interrelationships between context, identity, and purpose. Therefore, incorporating intersectionality in a meaningful way requires moving away from a binary understanding of identity that perpetuates false dichotomies. Rather, identity should be approached as fluid. For example, current literature on gender-based healthcare research often operationalizes gender, focusing on differences between men and women [17]. A meaningful intersectional application requires reframing of gender to increase understanding of its various intersections. Examples of such an approach include the Multi-Facet Gender and Health Model and Sterling’s dynamic systems theory [5]. Next, it requires interrogating the role of identities through an examination of power and inequality. This means theorizing and analyzing how power and inequality are fostered and perpetuated by certain systems [5]. In medical education, this must occur at the level of the individual, classroom, and healthcare environment. At the level of the individual, learners should interrogate their identities and experiences. At the classroom, reflection should include analysis on the interrelationship between the primary identity as a medical learner and secondary identities. At the level of the healthcare environment, reflection is further advanced to interrogate how learners’ identity is connected to and influences patients’ identity, the treatment relationship, and the healthcare team environment. To achieve this level of thinking, critical consciousness is a vital skill. Critical consciousness is understood as a “reflective awareness of differences in power and privilege and the inequities that are embedded in social relationships” [1].To develop this skill, learners must engage in cognitive and affective processes that include critical self-reflection and discourse [1].

#### Practical Implications: Centering Reflexivity

From a practical perspective, we must consider how and when intersectionality should be integrated into medical education and the benefits it has for medial learners. Our findings suggest that reflexivity should be centered during these considerations. Table 2 describes the ways intersectionality can be implemented and the associated benefits of such changes.

**Table 2:** Avenues of incorporating intersectionality.


TYPE OF CHANGE	TECHNIQUES	BENEFITS

Curriculum	Incorporate psychosocial perspective Discussion on social categories Interdisciplinary collaboration	Foster integrative thinking of disease Appreciate lived experience Greater patient empathy

Professional Competency	Incorporation of critical reflection and reflexivity Appraisal of systems of power	Understanding of self and other Professional Identity development

Structural Change	Accountability on the actions of educators and mentors Dismantling teaching hierarchies Identification of hidden curricular influences	Promote positive socialization processes Support safe learning spaces


Curriculum changes present a formalized approach for integrating intersectionality in medical education. It has the potential for fostering integrative thinking on psychosocial, biological, and pathophysiological manifestations of disease from an equity and identity focused lens [15,39]. For example, when teaching metabolic syndrome, discussions on disease processes must be rooted in consideration of associated risk factors. That includes conversations on non-health related contributing factors such as food security, social categories, and health access. To achieve a higher level of thinking students must be encouraged to understand the intersections of disease and social categories. This serves to improve how illness is appraised and understood, creating avenues for greater patient empathy [40].

At the same time, changes can also occur at the professional competency training level. Incorporation of critical reflection on systems of power can help learners better understand inequity and the circumstances and identities that contribute to it [39,41]. For example, discussions on Indigenous health from an intersectional perspective would include reflection on the role of colonialism and the influences of colonial powers to the appreciate the inherent power dynamics contributing to Indigenous oppression. Moreover, critical reflection on learner’s social categories and associated experiences can foster comprehensive self-understanding [42]. Prompting learners to consider who they are and the experiences that have formed them create avenues for appreciating the limits of our knowledge and identifying the assumptive beliefs we possess [41]. Both processes which contribute to comprehensive professional identity development of learners.

Lastly, the implementation of intersectional frameworks into the hidden curriculum presents an opportunity to limit the harms associated with the socialization process of becoming a healthcare professional. The hidden curriculum refers to a process outside of the formal curriculum that willingly or unwillingly transmits norms and values. This can include both positive and negative forms of learning [43]. The hidden curriculum has frequently been cited as an issue of contention for medical learners due to its contradictory undertones [44]. Intersectional thought presents an opportunity for educators and mentors to engage in self-accountability, ensuring their actions are consistent with the formalized teaching of learners. Moreover, it includes applying intersectional frameworks to minimize the power differentials between learners and educators, such that in instances of harm learners are empowered to speak up [44].

Ultimately, meaningful application of intersectionality in medical education requires integration of reflexivity, identity, and power. We must reflect on who we are and the experiences that have shaped us, analyse the influence of our identities and social context, and appreciate our connection to systems of privilege and disadvantage. With these appreciations, literature on intersectionality in medical education can then spark discourse on how to incorporate these principles into scholarly settings.

### Limitations

The results of this meta-narrative review must be considered within the context it was conducted. The analysis excludes dissertations, non-empirical research, and literature reviews. Moreover, the scope of our review focused on medical education, potentially explaining the limited literature available for analysis. Future studies may benefit from broadening the scope to include health professions education and including a more diverse sample of scholarly information. In addition, our attempts to seek peer review were not extensive and may have benefitted from broader consultation.

The limited number of articles on the topic of intersectionality and the gaps in translating knowledge into practice suggest that research on intersectionality in medical education is in its infancy. While conducting the meta-narrative review our team struggled to find relevant articles that met the outlined inclusion and exclusion criteria. Despite revisiting the process several times, there were limited examples that demonstrate debates, dilemmas, and dialogue in the literature. Thus, these meta-narratives may reflect limited investigation on the topic which can be alleviated as discourse evolves. We recognize that our search took place prior to the proliferation of EDI initiatives in medical education in 2020 and 2021, however, we believe that our findings provide an important window into a specific cross-section in history which took place up until 2020.

## Conclusion

Overall, our meta-narrative review highlights the complexity of intersectionality in medical education. Intersectionality has been used as a theory and framework to understand the relational and multiplex layers of identity to inform professional identity development for medical learners. It has also been used as an approach to understand systems of power aiding in identification of bias in healthcare spaces. Despite the flexibility in interpretation there is a contrasting narrative on the scope and purpose of intersectionality producing tensions. Concepts such as identity, categorization, and systems of power have been differently conceptualized in the literature. At the same time, limited research on intersectionality, simplification of intersectionality as a term for diversity, and superficial application compromises the depth and quality of intersectional research. Without understanding why intersectionality is used, how it is used, and when it can be used intersectional research in medical education will continue to be stagnant. Advancing discourse requires an interrogation of the intersectionality, its facets, and its role in advancing conversations on learning experiences, spaces, and processes.
